# LncRNA HOTAIR promotes DNA damage repair and radioresistance by targeting ATR in colorectal cancer

**DOI:** 10.32604/or.2024.044174

**Published:** 2024-07-17

**Authors:** HAIQING HU, HAO YANG, SHUAISHUAI FAN, XUE JIA, YING ZHAO, HONGRUI LI

**Affiliations:** 1 Department of Endoscopic Center, Peking University Cancer Hospital (Inner Mongolia Campus) & Affiliated Cancer Hospital of Inner Mongolia Medical University, Huhhot, 010020, China; 2 Department of Radiation Oncology, Peking University Cancer Hospital (Inner Mongolia Campus) & Affiliated Cancer Hospital of Inner Mongolia Medical University, Key Laboratory of Radiation Physics and Biology of Inner Mongolia Medical University, Peking University Cancer Hospital (Inner Mongolia Campus) & Affiliated Cancer Hospital of Inner Mongolia Medical University, Huhhot, 010020, China; 3 Graduate School, Inner Mongolia Medical University, Huhhot, 010020, China

**Keywords:** LncRNA HOTAIR, CRC, radioresistance, DNA damage repair, ATR

## Abstract

Long non-coding RNAs (lncRNAs) have been implicated in cancer progression and drug resistance development. Moreover, there is evidence that lncRNA HOX transcript antisense intergenic RNA (HOTAIR) is involved in colorectal cancer (CRC) progression. The present study aimed to examine the functional role of lncRNA HOTAIR in conferring radiotherapy resistance in CRC cells, as well as the underlying mechanism. The relative expression levels of HOTAIR were examined in 70 pairs of CRC tumor and para-cancerous tissues, as well as in radiosensitive and radioresistant samples. The correlations between HOTAIR expression levels and clinical features of patients with CRC were assessed using the Chi-square test. Functional assays such as cell proliferation, colony formation and apoptosis assays were conducted to determine the radiosensitivity in CRC cells with HOTAIR silencing after treatment with different doses of radiation. RNA pull-down assay and fluorescence *in situ* hybridization (FISH) were used to determine the interaction between HOTAIR and DNA damage response mediator ataxia-telangiectasia mutated- and Rad3-related (ATR). HOTAIR was significantly upregulated in CRC tumor tissues, especially in radioresistant tumor samples. The elevated expression of HOTAIR was correlated with more advanced histological grades, distance metastasis and the poor prognosis in patients with CRC. Silencing HOTAIR suppressed the proliferation and promoted apoptosis and radiosensitivity in CRC cells. HOTAIR knockdown also inhibited the tumorigenesis of CRC cells and enhanced the sensitivity to radiotherapy in a mouse xenograft model. Moreover, the data showed that HOTAIR could interact with ATR to regulate the DNA damage repair signaling pathway. Silencing HOTAIR impaired the ATR-ATR interacting protein (ATRIP) complex and signaling in cell cycle progression. Collectively, the present results indicate that lncRNA HOTAIR facilitates the DNA damage response pathway and promotes radioresistance in CRC cells by targeting ATR.

## Introduction

The incidence of colorectal cancer (CRC) is increasing steadily globally, especially in the young population [1]. CRC has been ranked third in cancer incidence and second in cancer-related mortality worldwide, thereby posing a major public health concern globally [2]. The 5-year survival rate for patients with early-stage CRC is >95% after treatment, with the possibility of being completely cured by surgery [3]. However, most patients with CRC are diagnosed at an advanced stage and the malignant progression of CRC compromises the efficacy of the treatment [4]. The current therapies implemented in the clinical management of patients with CRC include optimal surgical resection, chemotherapy and radiotherapy, with radiotherapy remaining a pivotal approach for treating locally advanced rectal cancer [5–8]. However, there is limited knowledge regarding the development of radioresistance and the underlying mechanisms in advanced CRC [5–7].

Noncoding RNAs (ncRNAs) are implicated in nearly all aspects of cellular and molecular processes in pathophysiological conditions including cancers [9]. NcRNAs consist of long noncoding RNAs (lncRNAs), circular RNAs (circRNAs) and microRNAs (miRNAs). LncRNAs are a class of ncRNA >200nt in length and their deregulation has been widely reported in different cancers [10]. Since the discovery of lncRNA in the 1990s, extensive evidence has demonstrated the functional engagement of lncRNAs in transcriptional control, post-transcriptional regulation and chromatin modification [11]. Recently, the knockdown of lncRNA TINCR was found to decrease the radioresistance in CRC cancer [12].

LncRNA HOX transcript antisense intergenic RNA (HOTAIR) gene is on chromosome 12 and it is in 2.2 kb length [13]. The dysregulation of HOTAIR was implicated in the malignant progression and drug resistance development in cancers, including lung cancer [14], breast cancer [15] and CRC [16]. Moreover, HOTAIR was found to be involved in DNA damage response (DDR) [17], a process that is activated to repair DNA upon the occurrence of DNA lesions induced by radiotherapy [18]. For instance, HOTAIR overexpression appeared to be linked to platinum resistance in ovarian cancer by sustaining DDR and the activation of nuclear factor kappa B (NF-κB) [19]. HOTAIR overexpression also was shown to promote DNA repair and radioresistance in breast and cervical cancers through different mechanisms [20,21]. By contrast, the inhibition of HOTAIR enhances radiosensitivity in pancreatic cancer cells by regulating autophagy [22].

In addition, ATR (ataxia-telangiectasia mutated- and Rad3-Related) was revealed as one of the key DDR sensing kinases that can be activated by DNA replication stress or DNA damage [23]. ATR was shown to form a stable complex with ATR interacting protein (ATRIP), playing a vital role in regulating the expression of DNA repair-related genes [24]. In lung cancer, the antisense lncRNA in the INK4 Locus (ANRIL) could promote DNA repair by stabilizing ATR, thereby enhancing radiotherapy resistance [25]. The ncRNA activated by DNA damage was also found to activate ATR/checkpoint kinase 1 (Chk1) signaling pathway to promote radioresistance in esophageal squamous cell carcinoma [26]. However, whether lncRNA HOTAIR also targets the DDR pathway to regulate radiosensitivity in CRC remains unclear.

The current study aimed to explore the functional role of lncRNA HOTAIR in conferring radiotherapy resistance to CRC cells and the underlying mechanism. The HOTAIR expression pattern was examined in radiosensitive and resistant CRC samples, and the correlation of its expression levels with the prognosis and clinical features was analyzed in patients with CRC. Through the *in vitro* and *in vivo* loss-of-function experiments, the role of HOTAIR in conferring the malignancy and radiosensitivity of CRC cells was further investigated. Finally, the present study established the potential engagement of the ATR/ATRIP DDR pathway in HOTAIR-dependent radiosensitivity regulation.

## Materials and Methods

### Patients’ recruitment and clinical specimen collection

A total number of 70 patients with CRC without prior chemotherapy or radiotherapy were recruited from November 2018 to May 2019. The CRC tumor and para-cancerous normal tissues were collected via surgery and stored at −80°C for further analysis. The patients were further administered adjuvant radiotherapy within 3 months after surgery. The survival of the patients was monitored for a period of 40 months. The patients with cancer recurrence after 1 year were deemed as radioresistant. The Ethics Committee of Peking University Cancer Hospital (Inner Mongolia Campus) and Affiliated Cancer Hospital of Inner Mongolia Medical University approved the present study and written informed consent was acquired from each enrolled participant.

### Cells and cell culture

Two human colorectal cancer cell lines (HCT116 and HT29) and human normal colorectal cells (FHC) were purchased from American Type Culture. HCT116 and HT29 cells were cultured in DMEM medium (Invitrogen, Carlsbad, CA, USA; Thermo Fisher Scientific, Inc., Waltham, MA, USA), whereas FHC cells were maintained in RPMI1640 medium (Procell Life Science & Technology Co., Ltd., Wuhan, China). Each medium was supplemented with 10% fetal bovine serum (Procell, Wuhan, China) and 100 U/ml penicillin/streptomycin (Procell, Wuhan, China). The cells were cultured in a humidified incubator at 37°C with 5% CO_2._

### Cell transfection

HCT116 and HT29 cells were seeded into a 6-well plate for 24 h until reaching 75% confluence. SiRNA targeting HOTAIR (si-HOTAIR) and negative control siRNA (si-NC) were obtained from Shanghai GenePharma Co., Ltd. (China). Cell transfection was conducted using Lipofectamine™ 2,000 (Invitrogen, Carlsbad, CA, USA; Thermo Fisher Scientific Inc., Waltham, MA, USA) according to the manufacturer’s instructions. The cells were harvested for functional experiments 48 h after the transfection. The following siRNA sequences were used: Si-NC, 5′-ATTGCGATTATCGGTGGAGGC-3′; Si-HOTAIR#1, 5′-GAGTGGCCCTGGGTGAATATT-3′; Si-HOTAIR#2, 5′-GTTCACACTGACCTCATTAAA-3′; and Si-HOTAIR#3, 5′-GAGACTTCTGGAAAGTAATAT-3′.

### Cell counting kit-8 (CCK-8) proliferation assay

HCT116 and HT29 cells were placed into 96-well plates at the density of 2,000 cells per well. Cells were cultured for 24, 48, 72, and 96 h, and at the indicated time point, 10 μl CCK8 reagent (Beyotime, Shanghai, China) was added to each well for 1 h incubation at 37°C. The absorbance was measured at 450 nm using a synergy HTX multimode microplate reader (BioTeK-Instruments, Winooski, VT, USA).

### Colony formation assay

To measure the colony formation activity, HCT116 and HT29 cells were seeded into a 6-well plate (1,000 cells/well) and the culture medium was changed every 3 days. After 14 days, cells were fixed with 4% paraformaldehyde at room temperature for 10 min and stained with 0.1% crystal violet (Beyotime, Shanghai, China) for 20 min. The number of colonies was counted using a Leica AM6000 microscope (Leica Microsystems GmbH).

### Flow cytometry analysis

For the apoptosis assay, cells subject to different treatments were trypsinized and washed twice with PBS, and 1 × 10^6^ cells were stained in 400 ul Annexin V and Propidium iodide (PI) staining solution (Annexin V-FITC/PI Apoptosis Kit; SIGMA-ALDRICH, Missouri, USA; Merck KGaA, Darmstadt, Germany) for 15 min in the dark. The percentage of apoptotic cells was detected using a BD FACS Canto™ II Flow Cytometer (BD Biosciences). For the cell cycle analysis, cells were washed with PBS and suspended at the density of 1 × 10^6^ cells/ml. A total of 5 ml cold 70% ethanol was added dropwise into the cell suspension to fix the cells at −20°C for 16 h, followed by PI staining (50 mg/ml) for 30 min at room temperature. The relative intensity of PI staining was detected using a BD FACS Canto™ II Flow Cytometer.

### RNA pull-down assay

Cells were transfected with biotinylated HOTAIR probes or NC probes (GenePharma, Shanghai, China). After 48 h, the cells were collected and incubated for 10 min in a specific pull-down lysis buffer (Ambion, Austin, TX, USA; Thermo Fisher Scientific Inc., Waltham, MA, USA). The lysate was incubated with 100 μl M-280 streptavidin beads (SIGMA-ALDRICH, Missouri, USA; Merck KGaA, Darmstadt, Germany) at 4°C for 4 h. The beads were washed for 4 times using pre-cooled lysis buffer. The bound RNA/protein samples were eluted using Laemmli buffer (Beyotime, Shanghai, China), and analyzed via western blot.

### Western blot

Protein samples from tissue or cell culture were harvested with RIPA lysis buffer with protease inhibitors (Shanghai Zeye Biotechnology Co., Ltd., Shanghai, China). A BCA protein assay kit (Beyotime, Shanghai, China) was used to quantify the concentration of each sample. An equal amount of protein was loaded into SDS-PAGE gel and separated before being transferred onto the PVDF membrane. The detection of target proteins was conducted using the following antibodies (Abcam, Cambridge, USA) at 4°C for 18 h: ATR (cat. no. ab2905, 1:1,000); RPA (cat. no. ab14460, 1:1500); CHK1 (cat. no. ab47574, 1:1,000); p-CHK1 (cat. no. ab58567, 1:1,000); CDC25A (cat. no. ab92892; 1:1,000); p-CDC25A (cat. no. ab182666; 1:1,000); CDK2 (cat. no. ab32147; 1:1,000); and GAPDH (cat. no. ab9485; 1:2,000). After washing with TBST buffer, the membrane was further probed with anti-mouse (cat. no. ab97023; 1:4,000) or anti-rabbit (cat. no. ab6721; 1:4,000) HRP conjugated secondary antibodies for 1 h at room temperature. The protein bands were developed using an enhanced chemiluminescence kit (Santa Cruz Biotechnology, Inc., Santa Cruz, CA, USA) and photographed on a GelDoc Go Gel Imaging System (Bio-Rad Laboratories, Hercules, CA, USA).

### RNA-immunoprecipitation (RIP) assay

The RIP kit (Millipore, Bedford, Massachusetts, USA) was used to detect the interaction between RNA and target protein. Cells were lysed by the cell lysis buffer containing protease inhibitor phenylmethylsulphonyl fluoride (PMSF) for 30 min on ice. After centrifuging at 14,000 rpm (4°C, 10 min), the supernatant was collected and incubated with Dynabeads Protein G conjugated with 4 μg anti-ATR antibody for 3 h at 4°C. Dynabeads were then washed three times using the lysis buffer, and the precipitated RNA samples were purified using Trizol reagent (Invitrogen, Carlsbad, CA, USA; Thermo Fisher Scientific Inc., Waltham, MA, USA), followed by cDNA reverse transcription and qRT-PCR analysis.

### Reverse transcription-quantitative (RT-q)PCR

RNA sample purification was conducted using Trizol reagent (Invitrogen, Carlsbad, CA, USA; Thermo Fisher Scientific Inc., Waltham, MA, USA) according to the manufacturer’s instructions. cDNA was reversed transcribed from a 1 μg RNA sample using the QuantiTect Reverse transcription kit (QIAGEN GmbH Valencia, CA, USA). QuantiNOVA SYBR Green PCR kit (QIAGEN GmbH Valencia, CA, USA) was used to perform quantitative PCR on a 7500 Real Time PCR System (Applied Biosystems, Waltham, MA, USA, Thermo Fisher Scientific Inc., Waltham, MA, USA). Afterward, the 2^–∆∆Cq^ method was used to analyze the relative expression levels of target genes and GAPDH was used as the internal reference. The following primers were used for the qPCR analysis (Sangon Biotech Co., Ltd. Shanghai, China): HOTAIR forward, 5′-CAGTGGGGAACTCTGACTCG-3’ and reverse, 5′-GTGCCTGGTGCTCTCTTACC-3′; GAPDH forward, 5′-TGACTTGAACCGCGACACCCA-3′ and reverse, 5′-CACCCTGTTGCTGTAGCCAAA-3′; U6 forward, 5′-GCTTCGGCAGCACATATACTAAAAT-3′ and reverse, 5′-CGCTTCACGAATTTGCGTGTCAT-3′; ATR forward, 5′-GGCCAAAGGCAGTTGTATTGA-3′ and reverse, 5′-GTGAGTACCCCAAAAATAGCAGG-3′.

### Fluorescence in situ hybridization (FISH)

The FISH experiment was conducted based on the previously described protocol [27]. HCT116 and HT29 cells were fixed in 4% paraformaldehyde for 15 min and treated with 1% pepsin solution. Cells were then incubated with 20 nM FITC-conjugated FISH probe to detect HOTAIR (GenePharma, Shanghai, China) in hybridization buffer at 73°C for 5 min. After washing, the cells were counter-stained with 100 nM DAPI (Beyotime, Shanghai, China) before imaging.

### Animal experiment

The xenograft model of CRC cells was established in Balb/c nude mice. HCT116 cells (2 × 10^6^) stably transfected with sh-HOTAIR or sh-NC were injected into the right flank of nude mice mouse. The animals were divided into four groups: i) sh-NC group, injected with sh-NC transfected cells; ii) sh-HOTAIR group, injected with sh-HOTAIR transfected cells; iii) sh-NC+IR group, injected with sh-NC transfected cells and treated with 8 Gy irradiation; and iv) sh-HOTAIR+IR group, injected with sh-HOTAIR transfected cells and treated with 8 Gy irradiation. Tumor volume was recorded every 3 days and calculated using the following formula: Volume = length × width^2^/2. At the end of week 4 after the treatment, tumor tissues were harvested for further analysis after the mice were euthanized by cervical dislocation. The animal study was approved by the Animal Care and Use Committee of the Peking University Cancer Hospital (Inner Mongolia Campus) and Affiliated Cancer Hospital of Inner Mongolia Medical University.

### Immunohistochemical (IHC) staining

Tumor tissues were fixed in 4% paraformaldehyde for 12 h and then sliced into 5-μm sections. After deparaffinization and hydration, antigen retrieval was conducted by heating the section in citrate unmasking solution at 95°C for 30 min. Afterward, the tumor section was incubated with PBS containing 2% H_2_O_2_ for 10 min at room temperature. The section was blocked for 1 h at room temperature with 5% normal goat serum (Beyotime, Shanghai, China) and then incubated with primary antibodies overnight: γH2AX (1:200; cat. no. ab11174; Abcam, Cambridge, USA), and TP53-binding protein 1 (53BP1; 1:250; cat. no. ab21083; Abcam, Cambridge, USA); followed by the labeling with HRP-conjugated secondary antibody (1:2,500; cat. no. ab6721; Abcam, Cambridge, USA) for 1 h at room temperature. The color development was conducted using a DAB IHC Detection kit (Beyotime, Shanghai, China). The images were captured under a Leica AM6000 microscope.

### Terminal deoxynucleotidyl transferase biotin-dUTP nick end labeling (TUNEL)

TUNEL cell apoptosis detection kit (Beyotime, Shanghai, China) was used for cell death staining in tissue sections. After deparaffinization and hydration, the sections were incubated with 20 μg/ml Dnase-free Protease K (Shanghai Zeye Biotechnology Co., Ltd., Shanghai, China) at 37°C for 15 min. After washing with PBS, the sections were incubated in 3% H_2_O_2_ solution at room temperature for 10 min. Afterward, two drops of TdT reaction mixture were added to the section for incubation at 37°C for 60 min. The sections were washed with PBS for 4 times and the nuclei were stained with 100 nM DAPI for 5 min. The images were captured under the Leica AM6000 microscope.

### Immunofluorescence

Cells were fixed with 4% formaldehyde at room temperature for 5 min, followed by permeabilization with 0.05% Triton-X100 (Beyotime, Shanghai, China). After the blocking with 5% normal goat serum for 1 h at room temperature, the cells were labeled with ATR antibody (1:500; H00000545A01; Abnova, Walnut, CA, USA) and ATRIP antibody (1:500; cat. no. ab245632; Abcam, Cambridge, USA) for 4 h at 4°C. After washing, the cells were incubated with goat anti-mouse IgG H&L (Alexa Fluor® 568; 1:1,500; cat. no. ab175473, Abcam, Cambridge, USA) and goat anti-rabbit IgG H&L (Alexa Fluor® 488; 1:2,000, cat. no. ab150077; Abcam, Cambridge, USA) for 1 h at room temperature. After the nuclear staining with DAPI, the images were captured under a Leica AM6000 microscope.

### Statistical analysis

All experiments were repeated 3 times and the data are displayed as mean ± standard deviation. GraphPad Prism 8.0 (GraphPad, San Diego, CA, USA) was used for the determination of statistical significance. Unpaired Students’ *t*-test was used for the comparison between two groups, whereas one-way ANOVA was adopted for multiple comparisons. Data from multiple time points were analyzed using two-way ANOVA. *p* < 0.05 was considered to indicate a statistically significant difference.

## Results

### HOTAIR expression is upregulated in CRC tissues

**Figure 1:**
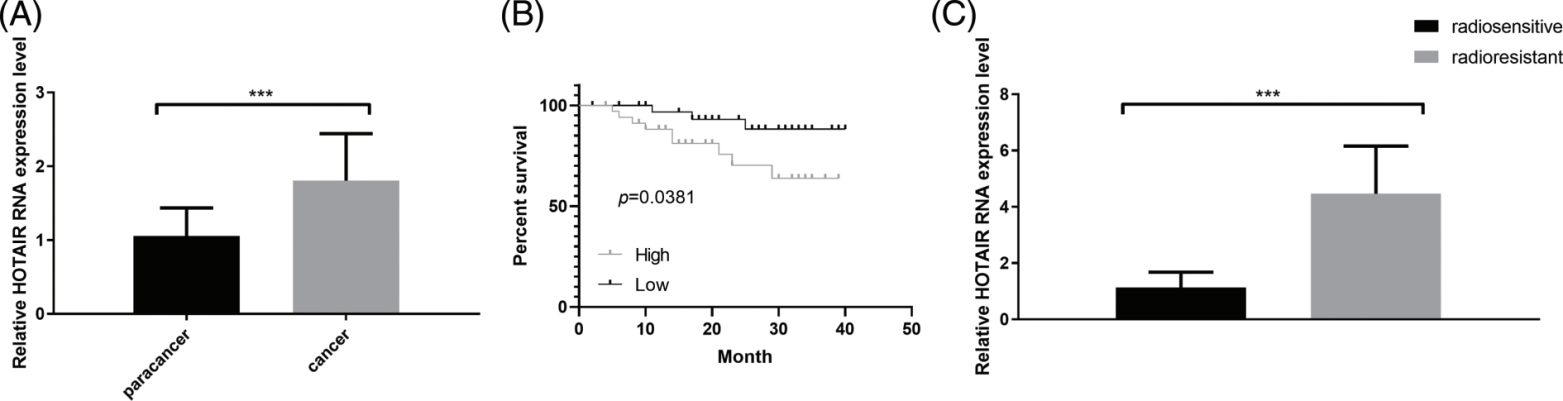
HOTAIR expression is upregulated in CRC tissues. (A) qRT-PCR was performed to detect the expression levels of HOTAIR in CRC tumors and adjacent normal tissues (n = 70 pairs). (B) Kaplan-Meier curve analysis of the overall survival in patients with CRC based on the expression of HOTAIR (n = 70, 35 cases in low or high-expression groups). (C) qRT-PCR detection of HOTAIR expression levels in radiosensitive (n = 28) and radioresistant (n = 42) CRC samples. ***p < 0.001.

A total of 70 pairs of CRC and adjacent normal tissues were collected to analyze HOTAIR expression via RT-qPCR. HORAIR levels were significantly increased in CRC tumor tissues compared with that in the normal counterparts (Fig. 1A). The patients were divided into high- and low- low-expression groups based on the median value of HORAIR levels. Kaplan-Meier survival curve analysis indicated that the overall survival was poorer in patients with CRC with high HOTAIR expression levels (Fig. 1 B). The above results suggested that the overexpression of HOTAIR may contribute to the poor prognosis in patients with CRC. Subsequently, the samples from patients who showed radioresistance (cancer recurrence 1 year after radiotherapy) and those who were sensitive to radiotherapy (no cancer recurrence 1 year after radiotherapy) were analyzed. Compared with the radiosensitive samples, HOTAIR was remarkably upregulated in the radioresistant CRC tissues, indicating that HOTAIR might contribute to the development of resistance to radiotherapy in CRC (Fig. 1C). In addition, the correlation between clinical features of 70 patients with CRC and HOTAIR expression levels were analyzed using the Chi-square test. A high level of HOTAIR was significantly associated with more advanced TNM grades and distance metastasis (Table 1). All these findings suggested that HOTAIR could be considered as a prognostic biomarker in CRC.

**Table 1:** Correlation between HOTAIR expression and clinicopathological parameters in patients with CRC

Characteristic	High expression (n = 35)	Low expression (n = 35)	*p*
Age			0.467
<60	19	22	
≥60	16	13	
Sex			0.225
Male	17	12	
Female	18	23	
Tumor size			0.056
<2	14	22	
≥2	21	13	
TNM stage			0.029
I–II	16	25	
III–IV	19	10	
Tumor differentiation			0.073
Well/moderate	25	31	
Poor	10	4	
Distant metastasis			0.034
M0	21	29	
M1	14	6	

### HOTAIR knockdown suppresses cell proliferation and promotes cell apoptosis in CRC cells

**Figure 2:**
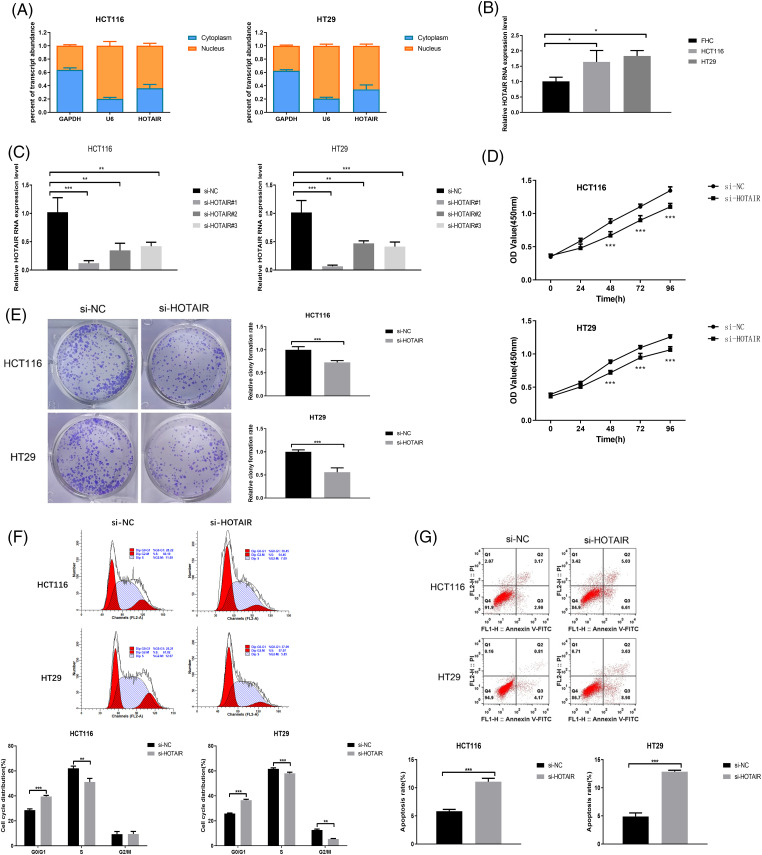
HOTAIR knockdown suppresses cell proliferation and promotes cell apoptosis in CRC cells. (A) Relative abundance of HOTAIR detected in the cytoplasmic and nuclear fractions of HCT116 and HT29 cells. (B) qRT-PCR detection of HOTAIR expression levels in CRC cell lines (HCT116 and HT29) and human colorectal mucosal cells (FHC). (C) qRT-PCR analysis of HOTAIR expression levels upon the transfection of si-NC (control siRNA) and siRNAs targeting HOTAIR (si-HOTAIR#1, #2, and #3). (D) CCK-8 cell proliferation assay, (E) Colony formation assay, (F) Cell cycle distribution, and (G) Apoptotic event detection in HCT116 and HT29 cells upon the transfection of si-NC or si-HOTAIR#1. *p < 0.05; **p < 0.01; ***p < 0.001.

To explore the biological function of HOTAIR in CRC cells, nuclear and cytoplasmic separation experiments were performed to measure the relative abundance of HOTAIR. HOTAIR showed a preferential localization in the nucleus when compared with the cytoplasmic compartment (Fig. 2A). HOTAIR also displayed a significant upregulation in HCT116 and HT29 CRC cells when compared with FHC (Fig. 2B). To reduce HOTAIR levels, CRC cells were transfected with three different siRNAs. Compared with the si-NC, si-HOTAIR#1 showed the strongest silencing effect, which was selected for the loss-of-function experiment (Fig. 2C). CCK-8 proliferation assay and colony formation experiment demonstrated that the silencing of HOTAIR via siRNA transfection significantly suppressed cell growth and colony formation in both HCT116 and HT29 cells (Figs. 2D and 2E). Furthermore, HOTAIR silencing enhanced the proportion of CRC cells in the G_0_/G_1_ phase and induced a significant increase in apoptotic events in both HCT116 and HT29 cells (Figs. 2F and 2G). Taken together, the present results suggested that HOTAIR could act as an oncogenic factor in CRC cells.

### Silencing HOTAIR promotes radiosensitivity in CRC cells

**Figure 3:**
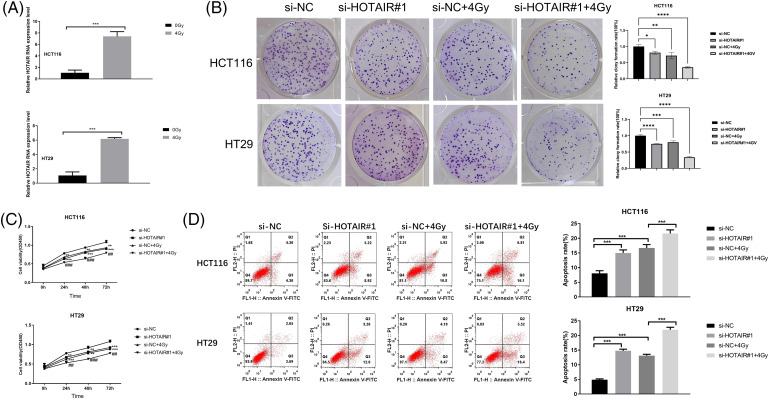
Silencing HOTAIR promotes radiosensitivity in CRC cells. (A) The detection of HOTAIR by qRT-PCR in HCT116 and HT29 cells treated with or without irradiation (4Gy). (B–D) CRC cells were transfected with si-NC or si-HOTAIR#1, and then treated with or without irradiation (4Gy). (B) Colony formation assay, (C) CCK-8 proliferation assay, and (D) Apoptosis analysis in the above conditions. *p < 0.05, **p < 0.01; ***p < 0.001; ****p < 0.0001; ^##^*p* < 0.01; ^###^*p* < 0.001.

To investigate the impact of HOTAIR knockdown on radiation sensitivity in CRC cells, HCT116 and HT29 cells were treated with or without irradiation (4Gy). It was found that HOTAIR expression levels were significantly increased 24 h after the irradiation (Fig. 3A). In the colony formation assay and CCK-8 proliferation assay, HOTAIR silencing not only impaired the cell proliferation in both HCT116 and HT29 cells but also aggravated the detrimental effect of irradiation on the cell growth (Figs. 3B and 3C). In addition, flow cytometry analysis demonstrated that HOTAIR knockdown also promoted the induction of apoptotic cells upon irradiation (Fig. 3D). These data suggested that silencing HOTAIR could promote radiosensitivity in CRC cells.

### HOTAIR silencing enhances the radiosensitivity of CRC tumors in vivo

**Figure 4:**
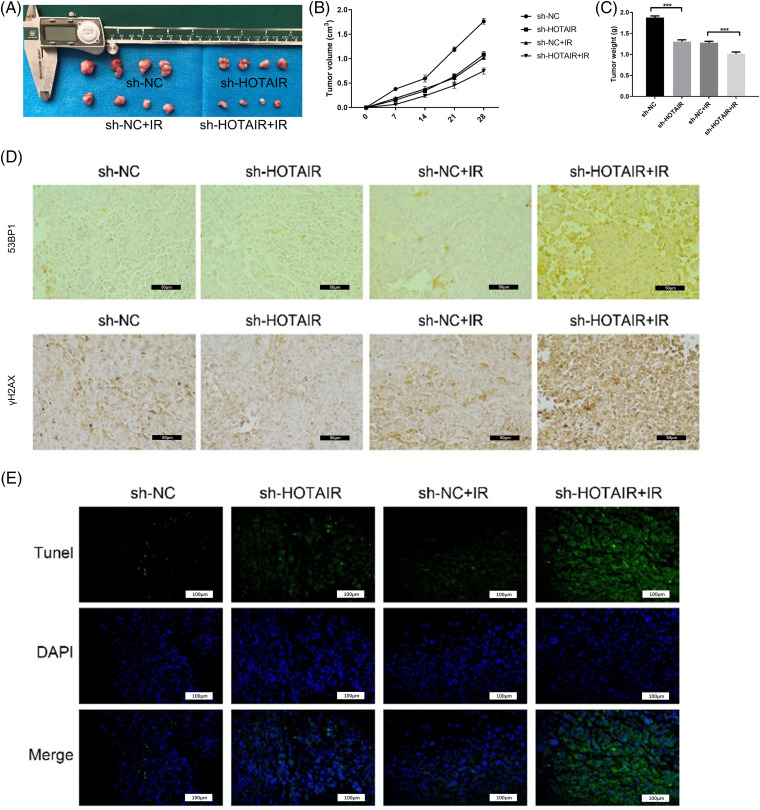
HOTAIR silencing enhances the radiosensitivity of CRC tumors *in vivo*. HCT116 cells with stable HOTAIR knockdown or control shRNA (sh-NC) were injected into the nude mouse as the subcutaneous tumor model. The mice were also administrated with three doses of 8Gy irradiation. (A) The images of tumor samples were harvested from nude mice. (B) The summary of tumor volume change. (C) The summary of tumor weight at day 28. (D) Immunohistochemistry (IHC) staining of γH2AX and 53BP1 in tumor tissues. (E) TUNEL analysis of apoptotic events in tumor tissues, Bar = 100 μm. ***p < 0.001.

To further corroborate the effect of HOTAIR on CRC radiotherapy *in vivo*, HCT116 cells with stable HOTAIR knockdown or transfected with sh-NC were injected into nude mouse to create a subcutaneous tumor model. The mice were also administrated with three doses of 8 Gy irradiation. After 4 weeks, the analysis of xenograft tumor samples showed that HOTAIR silencing attenuated tumor growth *in vivo*. The application of irradiation also suppressed the tumorigenesis of CRC cells in nude mice, and HOTAIR silencing synergized with irradiation treatment to suppress the tumor formation (Figs. 4A–4C). To understand the mechanism of radio sensitization, the present study investigated whether HOTAIR knockdown affects the formation of DNA damage foci (53BP1 and γH2AX staining). IHC staining showed that compared with the sh-NC group, HOTAIR silencing or irradiation treatment alone did not show an observable effect on γH2AX and 53BP1 staining in the tumor tissues; whereas the joint application of HOTAIR silencing and irradiation strongly increased the staining levels of γH2AX and 53BP1 (Fig. 4D). Furthermore, TUNEL staining of apoptotic events suggested that the number of apoptotic cells were significantly increased in the tumor samples after HOTAIR knockdown or irradiation treatment when compared with sh-NC groups; and the joint application of HOTAIR silencing and irradiation induced massive apoptotic events in the tumor tissues (Fig. 4E). Thus, HOTAIR knockdown could sensitize CRC tumors to irradiation and promote apoptosis induction upon irradiation.

### Interaction of HOTAIR and ATR in CRC cells

**Figure 5:**
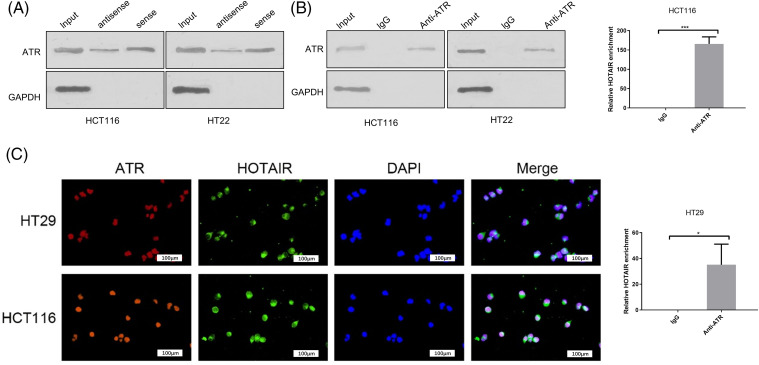
The interaction between HOTAIR and ATR in CRC cells. (A) RNA pull- down analysis of ATR and GAPDH using biotin-labeled HOTAIR probe. (B) RIP-PCR analysis of HOTAIR enrichment with anti-ATR antibody or IgG control. (C) Immunofluorescence and FISH analysis of ATR and HOTAIR in CRC cells. Bar = 100 μm. *p < 0.05; ***p < 0.001.

ATR is an important checkpoint protein in the cell cycle, which prevents cells from entering mitosis when cells are burdened with DNA damage [28]. Subsequently, the present study investigated whether HOTAIR interacts with ATR by performing an RNA-protein pull-down assay. Using GAPDH protein as the negative control, it was demonstrated that ATR protein could be precipitated by biotin-labeled HOTAIR probe in CRC cells (Fig. 5A). Furthermore, RIP-PCR analysis indicated that HOTAIR could be precipitated by anti-ATR antibody (Fig. 5B). FISH and ATR immunostaining analysis revealed the co-localization of ATR and HOTAIR in the nucleus of HCT116 and HT29 cells (Fig. 5C). These data demonstrated the interaction between ATR and HOTAIR.

### HOTAIR regulates radiosensitivity in CRC cells by targeting the DNA repair pathway

**Figure 6:**
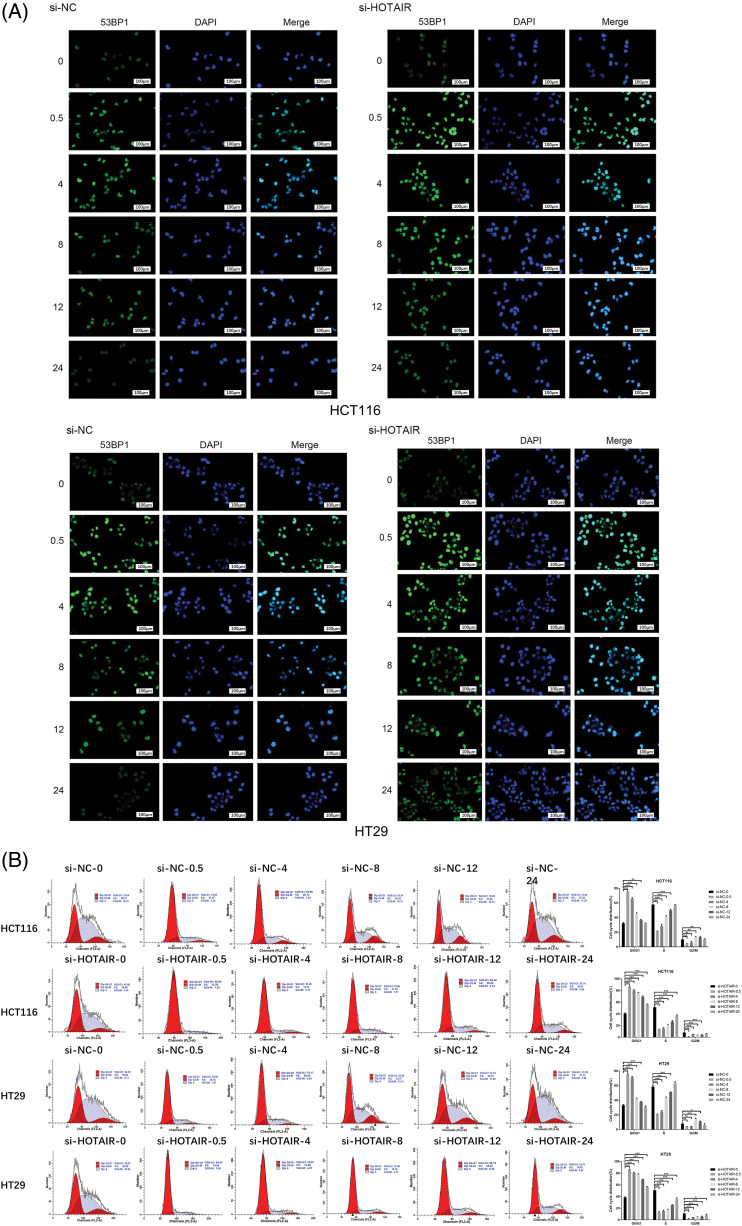
HOTAIR regulates radiosensitivity in CRC cells by targeting the DNA repair pathway. CRC cells transfected with si-NC or si-HOTAIR were treated with 4Gy irradiation. (A) cells were immunostained with 53BP1 at different time points (0, 0.5, 4, 8, 12 and 24 h) after irradiation. (B) Cell cycle analysis of CRC cells at different time points after irradiation. Bar = 100 μm. *p < 0.05; **p < 0.01; ***p < 0.001.

Since HOTAIR interacts with DNA damage sensor protein ATR, the present study investigated whether HOTAIR silencing could modulate DNA damage repair upon irradiation. 53BP1 DNA damage foci were detected in cells transfected with si-NC or si-HOTAIR at different time points after the irradiation (4 Gy). In cells with si-NC transfection, the levels of 53BP1 foci were increased at 0.5 and 4 h after the irradiation, then gradually decreased to the baseline level after 24 h (Fig. 6A). However, in the cells with HOTAIR knockdown, the intensity of 53BP1 staining remained at a relatively higher level after irradiation when compared with the cells transfected with si-NC (Fig. 6A), indicating persistent DNA damages in cells with HOTAIR silencing. In addition, cell cycle analysis showed that irradiation-induced G_0_/G_1_ arrest was gradually released in the control cells after 24 h; however, in the HOTAIR silencing group, there were relatively higher levels of cells being arrested in G_0_/G_1_ phase after 24 h (Fig. 6B). Therefore, these data suggested that HOTAIR could regulate radiosensitivity in CRC cells by targeting DNA repair pathway.

### Silencing HOTAIR affects the ATR-ATRIP complex and signaling in cell cycle progression

**Figure 7:**
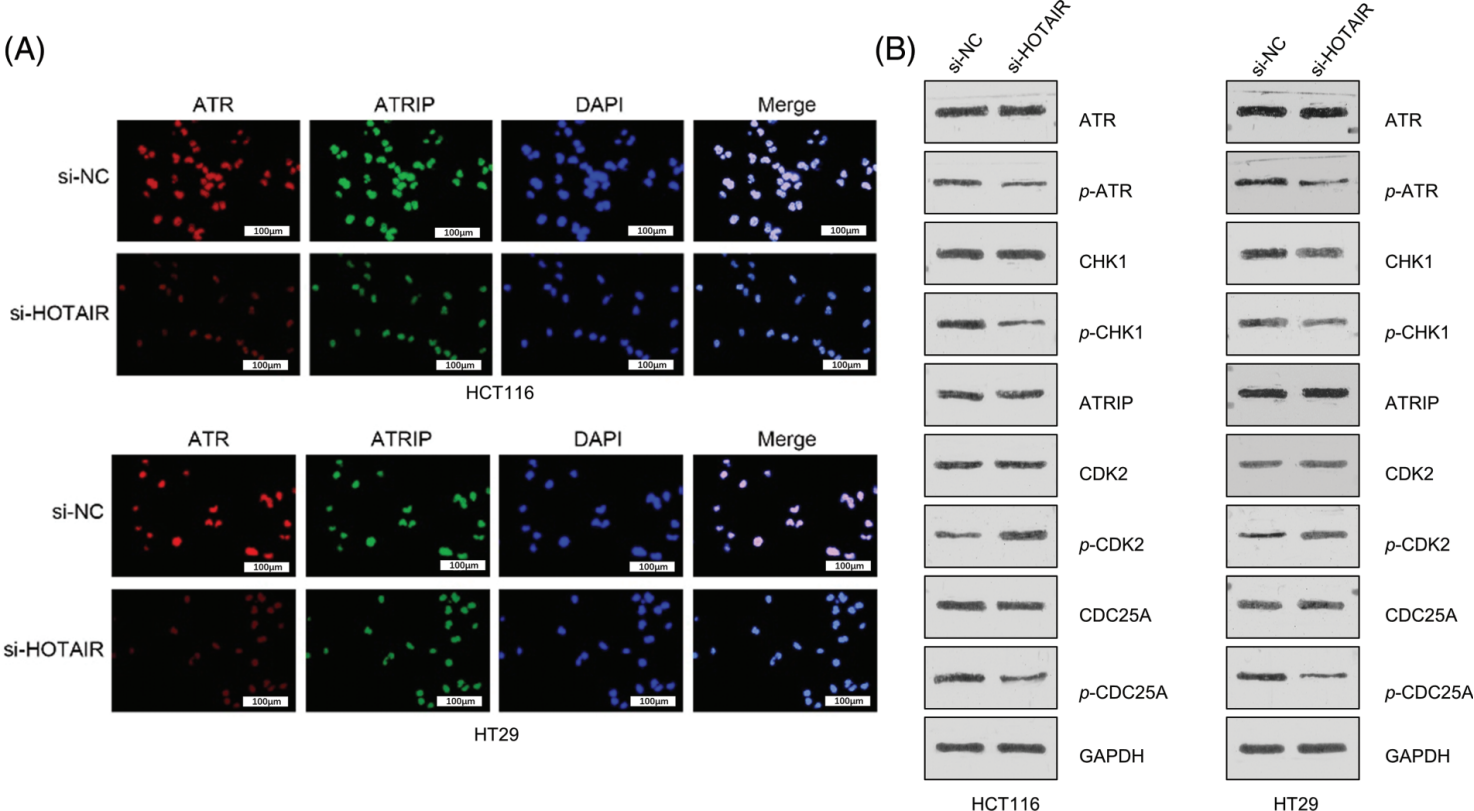
Silencing HOTAIR affects ATR-ATRIP complex and signaling in cell cycle progression (A) Co-immunostaining of ATR and ATRIP in CRC cells with or without HOTAIR knockdown after 4Gy irradiation. (B) Western blot analysis of the phosphorylation levels of DNA damage sensors (CHK1 and ATR) and cell cycle checkpoint proteins (CDK2 and CDC25A). Bar = 100 μm.

To better understand how HOTAIR influences DDR and cell cycle progression, co-immunostaining of ATR and ATRIP was performed in CRC cells with or without HOTAIR knockdown. ATR and ATRIP signaling showed nuclear co-localization upon irradiation (4 Gy), and silencing HOTAIR reduced the nuclear signals of ATR and ATRIP (Fig. 7A). DDR activation was further examined by analyzing the phosphorylation level of ATR and CHK1, as well as the cell cycle checkpoint proteins CDK2 and CDC25A. HOTAIR silencing suppressed the phosphorylation of ATR and CHK1, indicating the impairment of DDR. In the meanwhile, HOTAIR silencing reduced the phosphorylation of CDC25A, but promoted the inhibitory phosphorylation of CDK2 (Fig. 7B). However, the total protein levels of ATR and ATRIP remained unchanged after HOTAIR silencing. These data suggested that silencing HOTAIR could impair ATR-ATRIP complex and signaling in cell cycle progression.

## Discussion

The present study demonstrated that HOTAIR was overexpressed in CRC tumor tissues, especially in radioresistant tumor samples. The elevated expression of HOTAIR was correlated with a poor prognosis in patients with CRC. Silencing HOTAIR promoted apoptosis and radiosensitivity in CRC cells, and also attenuated the tumorigenesis of CRC cells and enhanced the sensitivity to radiotherapy in a mouse xenograft model. Mechanistically, HOTAIR could interact with ATR to regulate DNA damage repair. These findings indicated HOTAIR as an oncogenic factor promoting radioresistance in CRC progression, thereby targeting HOTAIR could be employed as an intervention strategy to promote radiosensitivity.

There is an increasing prevalence of CRC worldwide and neoadjuvant radiotherapy remains a major treatment after surgical resection [29]. Generally, neoadjuvant radiotherapy shows clinical benefits in improving the survival chance and reducing the risk of cancer recurrence [30]. However, a fraction of patients with CRC suffer from cancer relapse after radiotherapy, suggesting the development of radioresistance. Accumulating evidence indicates that the dysregulation of ncRNAs, such as lncRNAs, may contribute to the development of radioresistance in cancers and could serve as biomarkers for predicting radioresistance and survival [31]. The present study showed that HOTAIR upregulation was associated with radioresistance in patients with CRC and its overexpression was correlated with poor prognosis, indicating the prognostic potential of HOTAIR in CRC progression. Previous studies also demonstrated that HOTAIR overexpression promotes DNA repair and radioresistance in breast and cervical cancers through different mechanisms [20,21]. By contrast, the HOTAIR downregulation was shown to enhance radiosensitivity in pancreatic cancer and CRC [22,32,33]. In addition, the present results indicated that silencing HOTAIR enhanced the radiosensitivity of CRC cells and the sensitivity to radiotherapy in a xenograft mouse model. Thus, the present data and previous studies support the notion that HOTAIR functions as a tumor-promoting factor to conferring radioresistance in cancer progression.

It was further shown that HOTAIR interacts with DNA damage sensor ATR in CRC cells, which is critical for the suppression of cell cycle and initiation of DNA repair. Silencing HOTAIR caused the persistence of DNA damage markers (53BP and γH2AX) after irradiation treatment in CRC cells, suggesting an impaired DNA repair process. Radiation exposure in radiotherapy primarily causes DNA damage, and the cells with damaged DNA arrest the cell cycle and activate the cascade of DNA repair signaling pathways [1]. The balance between DDR activation and DNA damage repair affects the consequence of cell fate after radiation damage, and altering DNA damage repair can improve the efficacy of radiation therapy in cancers [34,35]. The accumulation of γH2AX and 53BP1 at damaged DNA is the hallmark of DDR activation, which is also required for DNA damage repair [36,37]. Moreover, γH2AX was also recognized as a radiosensitivity indicator in lung cancer [38] and CRC [39]. ATR is a DNA damage-sensing kinase that coordinates cell cycle progression and DNA damage repair. ATR can phosphorylate hundreds of downstream proteins, including CHK1 and γH2AX, to mediate DNA damage repair [39,40]. ATR-activated phosphorylation cascade leads to P53 phosphorylation and the stabilization of P53 results in cell cycle arrest [41]. Indeed, upon HOTAIR silencing, apart from the reduced phosphorylation of CHK1, the phosphorylation level of CDC25A (CDC25A functions to regulate the G_1_/S checkpoint by activating cyclin E/CDK2 kinase) was also reduced. Since CDC25A is a phosphatase that removes inhibitory phosphorylation in CDK2 [42], the reduced CDC25A phosphorylation and elevated CDK2 phosphorylation upon HOTAOR may account for the cell cycle arrest at the G_1_ phase.

The observed effects of HOTAIR in regulating DDR and DNA repair are also consistent with previous studies. For instance, a previous study showed that HOTAIR overexpression is linked to platinum resistance in ovarian cancer by sustaining DDR [19]. Qian et al. [21] revealed that HOTAIR overexpression promoted the expression of DNA repair factors, including DNA-protein kinases and Ku protein (Ku70 and Ku80). Gao et al. [43] reported that there was a positive correlation between HOTAIR expression level and the degree of DNA damage in individuals exposed to polycyclic aromatic hydrocarbons (PAHs), indicating that HOTAIR is implicated in modulating DDR induced by PAH exposure. Although previous studies also suggested the role of HOTAIR in dictating radiosensitivity in CRC [31,32], the present study provided novel insights into the molecular mechanism of HOTAIR-dependent radioresistance. Overall, the present data suggested the involvement of HOTAIR in regulating DDR and DNA repair, highlighting the possibility of targeting HOTAIR as a strategy to modulate radiosensitivity in CRC.

In summary, it was demonstrated that HOTAIR plays a pivotal role in dictating radioresistance and cancer progression in CRC and silencing HOTAIR promoted radiosensitivity *in vitro* and *in vivo*. It was further shown that HOTAIR interacted with DDR sensor ATR to modulate DNA repair and cell cycle progression upon irradiation. The current study suggests that targeting HOTAIR can be employed as a strategy to modulate radiosensitivity in CRC radiotherapy.

## Data Availability

The data is available upon reasonable request to corresponding author via email.
